# Adrenergic receptors inhibit TRPV1 activity in the dorsal root ganglion neurons of rats

**DOI:** 10.1371/journal.pone.0191032

**Published:** 2018-01-05

**Authors:** Yumi Matsushita, Miki Manabe, Naoki Kitamura, Izumi Shibuya

**Affiliations:** Laboratory of Veterinary Physiology, Faculty of Agriculture, Tottori University, Tottori, Japan; Hokkaido Daigaku, JAPAN

## Abstract

Transient receptor potential vanilloid type 1 (TRPV1) is a polymodal receptor channel that responds to multiple types of stimuli, such as heat, acid, mechanical pressure and some vanilloids. Capsaicin is the most commonly used vanilloid to stimulate TRPV1. TRPV1 channels are expressed in dorsal root ganglion neurons that extend to Aδ- and C-fibers and have a role in the transduction of noxious inputs to the skin into the electrical signals of the sensory nerve. Although noradrenergic nervous systems, including the descending antinociceptive system and the sympathetic nervous system, are known to modulate pain sensation, the functional association between TRPV1 and noradrenaline in primary sensory neurons has rarely been examined. In the present study, we examined the effects of noradrenaline on capsaicin-evoked currents in cultured dorsal root ganglion neurons of the rat by the whole-cell voltage clamp method. Noradrenaline at concentrations higher than 0.1 pM significantly reduced the amplitudes of the inward capsaicin currents recorded at –60 mV holding potential. This inhibitory action was reversed by either yohimbine (an α_2_ antagonist, 10 nM) or propranolol (a β antagonist, 10 nM). The α_2_ agonists, clonidine (1 pM) and dexmedetomidine (1 pM) inhibited capsaicin currents, and yohimbine (1 nM) reversed the effects of clonidine. The inhibitory action of noradrenaline was not seen in the neurons pretreated with pertussis toxin (100 μg/ml for 24 h) and the neurons dialyzed intracellularly with guanosine 5’- [β-thio] diphosphate (GDPβS, 200 μM), the catalytic subunit of protein kinase A (250 U/ml) or okadaic acid (1 μM). These results suggest that noradrenaline directly acts on dorsal root ganglion neurons to inhibit the activity of TRPV1 depending on the activation of α_2_-adrenoceptors followed by the inhibition of the adenylate cyclase/cAMP/protein kinase A pathway.

## Introduction

Pain sensation is important for animals to protect their bodies from harmful situations both inside and outside of the body. In vertebrates, nociceptive inputs to the skin are received by peripheral sensory nerve fibers extending from cell bodies in the dorsal root ganglion (DRG), and nociceptive signals are carried to the second-order neurons in the spinal dorsal horn by the conduction of action potentials through primary afferent fibers. One of the polymodal non-selective cation channels, transient receptor potential vanilloid type 1 (TRPV1), expressed on the membranes of nociceptive Aδ- and C-fibers, acts as a sensor of various types of stimuli, such as heat, acid, mechanical pressure and vanilloids. TRPV1 channels have a role in the first step of nociception [1–6].

On the other hand, the bodies of animals have mechanisms to relieve pain. The most significant system is the descending antinociceptive system (DAS) in the central nervous system (CNS). Among neurotransmitters that contribute to the DAS, noradrenaline (NA) is considered one of the most important transmitters and has significant roles in the spinal cord [7–11]. In the CNS, noradrenergic neurons whose cell bodies are located in the locus coeruleus in the pons extend their axons along the spinal cord and project around sensory neurons in the spinal dorsal horn [12–15]. The inhibitory action of noradrenergic neurons on sensory neurons is considered to result in pain relief [10]. In peripheral tissues outside of the spinal cord, the major origins of NA are the sympathetic nerve and the adrenal medulla. In addition to the analgesic actions of NA in the spinal cord, the injection of NA into artificially inflamed tissues reportedly reduces pain responses to mechanical noxious stimuli [16], and the administration of clonidine, an antagonist of the α_2_-adrenoceptor, to injured sciatic nerves reduces responses to noxious mechanical stimuli [17]. In contrast, NA injected under the skin reportedly enhances the pain sensation evoked by heat in human [18]. The physiological roles of the noradrenergic system in the regulation of nociception seem to be complicated, and understanding the underlying mechanisms completely is difficult.

Since all subtypes (α_1_, α_2_ and β) of adrenoceptors are expressed in DRG neurons [19–23], the actions of NA may affect TRPV1 activity. However, few studies have been conducted to elucidate the functional interaction between adrenoceptors and TRPV1. Very recently, α_2_-adrenoceptors have been reported to reduce the activity of TRPV1 in DRG neurons, and this effect is caused by the potentiation of calmodulin-dependent kinase II (CAMKII) activity [24]. Moreover, this action of α_2_-adrenoceptors was demonstrated to reduce capsaicin-evoked neurotransmitter release from the spinal terminals of primary sensory neurons, and this phenomenon was proposed to play a role in the analgesic action of the adrenergic system. However, since adrenergic drugs injected peripherally also show analgesic effects, other mechanisms may contribute to adrenergic system-mediated analgesia. In the present study, we examine the inhibitory action of NA on TRPV1 in the DRG neurons of the rat and identify another mechanism.

## Materials and methods

### Cell isolation and culture

All animal experiments were performed in accordance with the guidelines of Tottori University and this study is approved by the Institutional Animal Care and Use Committee, Tottori University. DRG neurons were isolated from adult male Wistar rats (7–10 weeks old) using procedures that have been reported previously [25–27]. The rats were sacrificed by decapitation under anesthesia with isoflurane, and all efforts were made to minimize the suffering of the rats. Ganglia were dissected from the entire length of the vertebral column. The collected ganglia were incubated at 37°C in Ca^2+^- and Mg^2+^-free phosphate-buffered saline (PBS) containing collagenase (Type IV, Worthington Biochemical; 400 U/ml) and DNase I (5 μg/ml; Sigma, St Louis, MO, USA) for 2 h and were then rinsed with PBS to remove the enzymes. Next, the ganglia were incubated in PBS containing trypsin (0.25% w/v; Invitrogen, Carlsbad, CA, USA) and DNase I (5 μg/ml) for 10 min. After enzymatic digestion, the cells were gently agitated with a silicon-coated Pasteur pipette and centrifuged to remove the enzymes. The isolated cells were suspended in Dulbecco’s modified Eagle medium (DMEM) with L-glutamine (584 mg/l), glucose (4.5 g/l) and phenol red (15 mg/l) and plated on coverslips. The cells were cultured at 37°C in a humidified atmosphere of 95% air and 5% CO_2_ until use. DMEM was supplemented with 10% fetal bovine serum (Biosera, St Kansan, MO, USA), 100 U/ml penicillin (Sigma), 100 ng/ml streptomycin (Sigma), and 5 μM cytosine β-D-arabinoside (Sigma). The culture medium was changed every 2 days. The neurons were used in the electrophysiological experiments after 3–7 days of culture.

### Electrophysiology

The whole-cell voltage clamp recording was conducted at room temperature (22–24°C). Heat-polished glass electrodes with a tip resistance of 2.5–4 MΩ were used. DRG neurons with a small diameter (10–30 μm) that had been reported to predominantly express TRPV1 [3, 28] were used to record the membrane currents. The normal bath solution consisted of (in mM): 154 NaCl, 6 KCl, 1.2 MgCl_2_, 2.5 CaCl_2_, 10 D-glucose and 10 HEPES and the pH was adjusted to 7.4 with Tris^+^. The pipette solution consisted of (in mM): 10 Na-gluconate, 130 K-gluconate, 4.5 MgCl_2_, 0.74 CaCl_2_, 10 EGTA-2K and 10 HEPES and the pH was adjusted to 7.3 with Tris^+^. The neurons were continuously perfused with the bath solution at a flow rate of 1–1.5 ml/min throughout the experiments. Currents were measured with the patch clamp amplifiers (Axopatch 200A; Molecular Devices, Sunnyvale, CA, USA or EPC-10; HEKA Electronik, Germany). The protocol to measure the current responses to capsaicin was performed at –60 mV holding potential and we usually applied capsaicin at 1 μM for 30 s. The cells that showed current responses greater than 100 pA to 1 μM capsaicin were used for the analysis.

### Drugs

Concentrated stock solutions of NA at 1 mM and guanosine 5’- [β-thio] diphosphate trilithium salt (GDPβS) at 40 mM were made by dissolving in distilled water. A concentrated stock solution of capsaicin at 1 mM was made by dissolving in DMSO. Concentrated stock solutions of the catalytic subunit of protein kinase A (cPKA) at 500 U/ml and okadaic acid at 1 μM were made by dissolving in the pipette solution. All of the stock solutions were stored at –30°C until use.

### Data analysis

**Fig 1:**
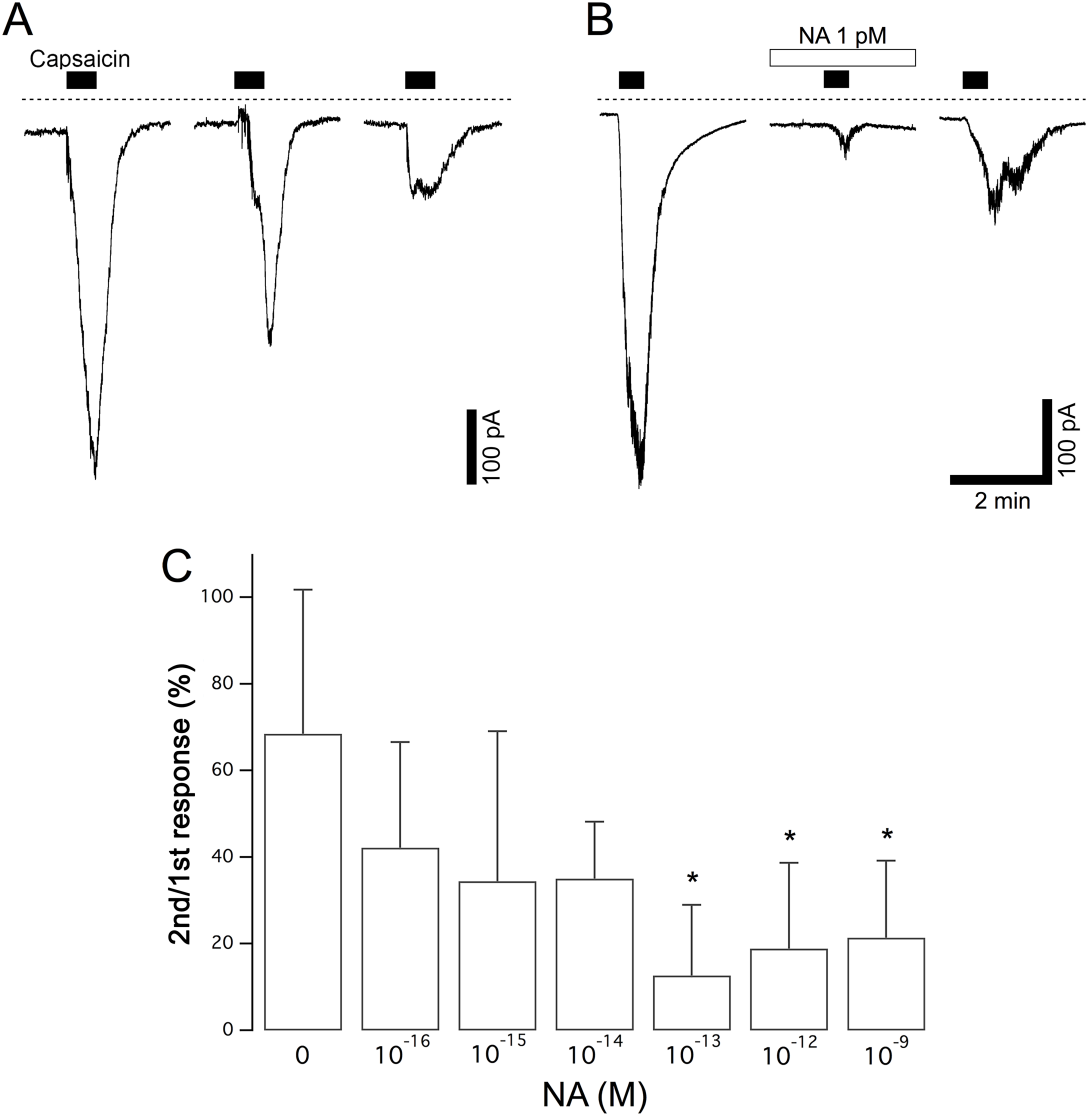
Inhibition of capsaicin currents by NA. (A) Control responses to capsaicin in the absence of NA. Capsaicin (1 μM) was applied for 30 s three times at intervals of 15 min to the same neuron. (B) The second stimulation by capsaicin was applied in the presence of NA (1 pM). NA was applied 2 min before the second capsaicin stimulation for 4.5 min. Dashed lines in panels A and B indicate zero current level. (C) The dose dependence of the inhibition of capsaicin currents by NA. The relative peak amplitudes of the capsaicin currents evoked by the second stimulation compared to those evoked by the first stimulation in each neuron are shown by the columns (2nd/1st response). The columns and vertical bars indicate the mean ± SD (n = 5, 6, 5, 5, 6, 8 and 5 from the left). * P < 0.05 against the control data in the absence of NA.

Data acquisition was performed at a sampling frequency of 10 kHz throughout the experiments by a personal computer (Macintosh; Apple, Cupertino, CA, USA) in conjunction with an analog/digital converter (Power Lab; AD Instruments, Castle Hill, NSW, Australia). The data were acquired and analyzed with Lab Chat (AD Instrument), Patch Master (HEKA), IGOR Pro (Wavemetrics, Redmond, WA, OR, USA) and Excel (Microsoft, Redmond, WA, USA). The data are presented as the mean values ± SEM (n = the number of observations), except in Fig 1 (mean ± SD). Statistical significance was assessed by Student’s *t*-test or ANOVA in accordance with the situation. Differences were considered statistically significant if P < 0.05.

## Results

### Inhibition of the capsaicin-induced currents by NA

At the beginning of each recording, the cells were voltage clamped at –60 mV, and depolarizing voltage steps were applied to the evoked currents through voltage-gated Na^+^ and K^+^ currents to confirm that a focused cell was a functional neuron. We analyzed current responses in the neurons that required holding currents less than –100 pA to be voltage clamped at –60 mV in this study. Under the voltage-clamped condition, capsaicin at 1 μM was applied for 30 s, and the application was repeated 3 times at an interval of 15 min in each neuron. In many DRG neurons, a transient inward current was evoked by the application of 1 μM capsaicin. The amplitudes of the capsaicin current reached their maximum peaks within 20 s and gradually decreased. The neurons that showed a current response larger than 100 pA in response to the first application of capsaicin were used for the analysis in this study. The amplitudes of the current responses to trains of stimulations with capsaicin gradually decreased because of the desensitizing property of TRPV1 channels. To assess the effects of agents on TRPV1 activities quantitatively, we calculated the ratio of the peak amplitude of the second responses to that of the first responses. The mean relative amplitude of the second capsaicin responses in the control experiments without any drugs other than capsaicin was 68.5 ± 14.9% (n = 5). To assess an effect of NA on the capsaicin current, NA was applied 2 min prior to the second stimulation with capsaicin and was continuously applied until 2 min after the removal of capsaicin. In the neuron shown in Fig 1B, the amplitude of the capsaicin current was greatly reduced by 1 pM NA. The amplitudes of the capsaicin currents in the presence of NA at each concentration varied greatly. For example, they ranged between 0 and 84.4% in the presence of NA at 1 fM. The summarized dose-dependent responses to NA, including these variations, are shown in Fig 1C. Significant inhibitory effects were observed in the presence of NA at concentrations higher than 10^−13^ M. The maximum inhibition ratio was 87.3 ± 16.2% (n = 6) at 10^−13^ M.

### Effects of specific antagonists of adrenoceptors

**Fig 2:**
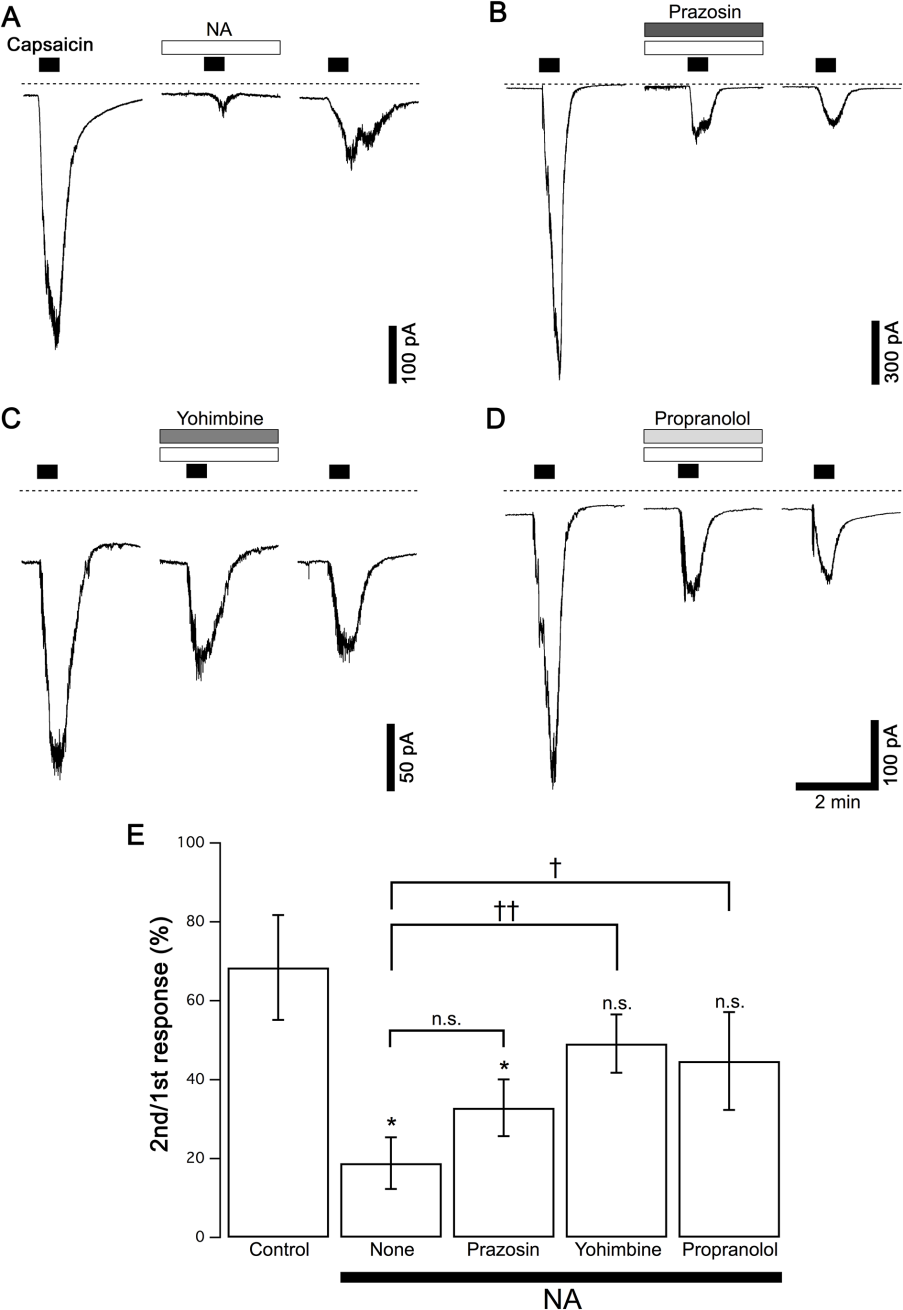
Effects of adrenoceptor antagonists on the NA-induced suppression of capsaicin currents. (A) Typical control responses to NA (1 pM) in the absence of antagonists are shown (Same traces as Fig 1B). (B-D) Prazosin (10 nM), yohimbine (10 nM) and propranolol (10 nM) were applied in combination with NA (1 pM) in the same protocol as A. Dashed lines in panels A-D indicate zero current level. (E) The relative peak amplitudes of capsaicin currents by the second stimulation compared to those by the first stimulation in each neuron are shown (2nd/1st response). The columns and vertical bars indicate the mean ± SEM (n = 5, 8, 5, 6 and 5 from the left). * P < 0.05 against the control data in the absence of NA; † P < 0.05, †† P < 0.01 against the control data in the absence of antagonists.

To determine which adrenoceptor subtype contributes to the inhibition of capsaicin currents in DRG neurons, we examined the effects of three antagonists of α_1_, α_2_ and β receptors on the inhibitory action of NA. Similar to the protocol in Fig 1, capsaicin was applied 3 times in each neuron, and the antagonists were applied with NA prior to the second capsaicin application. The typical current responses in the presence of prazosin (10 nM, an α_1_ antagonist), yohimbine (10 nM, an α_2_ antagonist) and propranolol (10 nM, a β antagonist) are shown in Fig 2B, 2C and 2D, respectively. Control responses (Fig 2A) were obtained from neurons in the same culture. The pooled data of the relative amplitudes of the current responses to the second capsaicin stimulation are shown in Fig 2E. In the presence of prazosin, NA inhibited the capsaicin current as much as in the absence of antagonists. In contrast, yohimbine and propranolol reversed the inhibition of capsaicin currents by NA, and NA did not inhibit the capsaicin current in the presence of yohimbine or propranolol (Fig 2E). These results suggest that α_2_ and/or β adrenoceptors contribute to the inhibitory action of NA on capsaicin currents.

### Effects of α_2_ and β agonists

**Fig 3:**
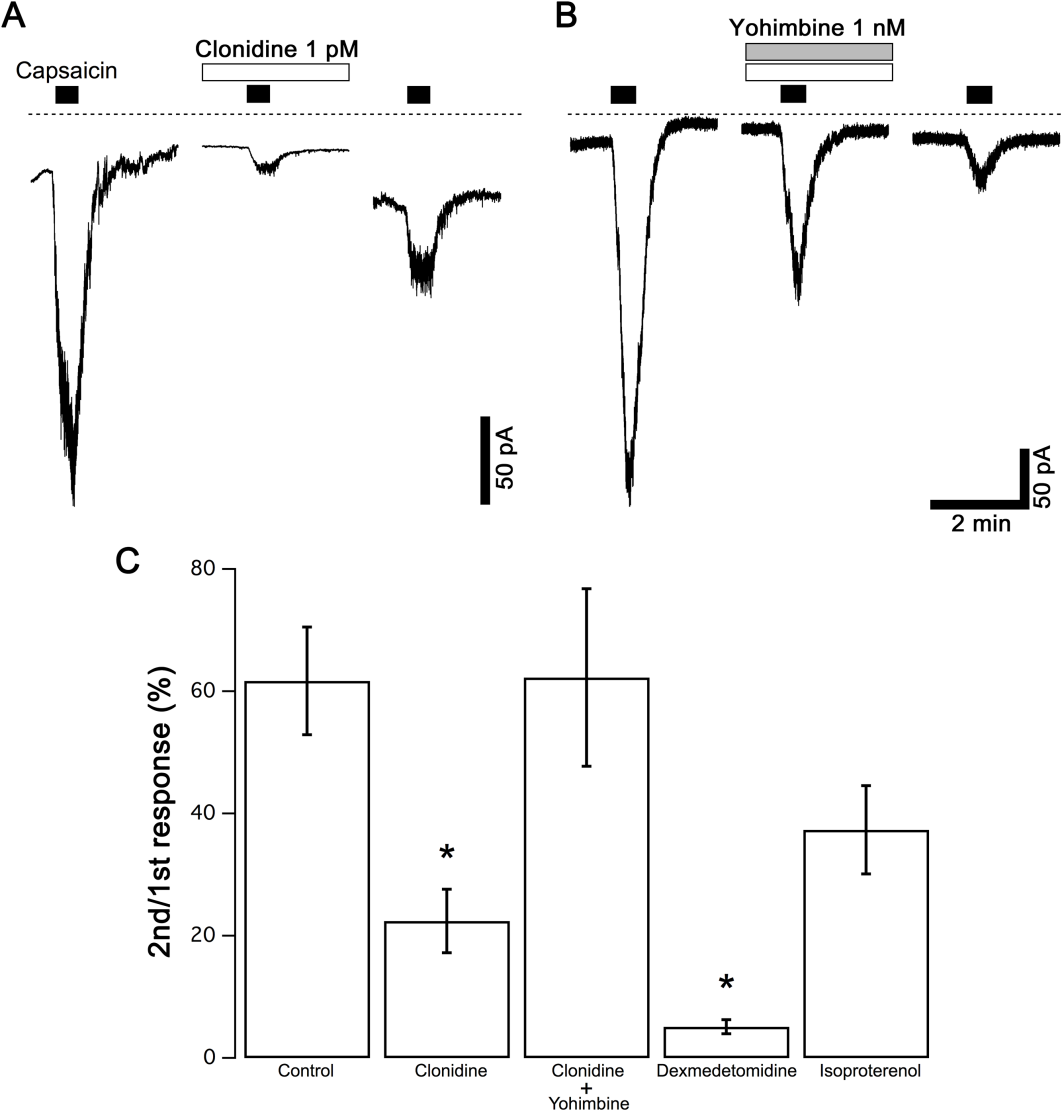
Effects of adrenoceptor agonists on capsaicin currents. (A) Typical responses to capsaicin (1 μM) in the absence and presence of clonidine (1 pM) are shown. (B) Yohimbine (1 nM) was applied in combination with clonidine (1 pM) in the same protocol as A. Dashed lines in panels A, B indicate zero current level. (C) The relative peak amplitudes of capsaicin currents by the second stimulation compared to those by the first stimulation in each neuron are shown (2nd/1st response). Amplitudes of capsaicin currents in the presence of dexmedetomidine (1 pM) and isoproterenol (1 pM) are also shown. The columns and vertical bars indicate the mean ± SEM (n = 9, 10, 5, 4 and 4 from the left). * P < 0.05 against the control data in the absence of adrenergic agonists.

The effects of clonidine, an α_2_-adrenoceptor agonist, and isoproterenol, a β adrenoceptor agonist, on capsaicin currents were examined because the α_2_ and β adrenoceptor antagonists were effective. Instead of NA, clonidine (1 pM), dexmedetomidine (1 pM) or isoproterenol (1 pM) was applied to the neurons in the same protocol as Fig 1. Clonidine (Fig 3A and 3C) and dexmedetomidine (Fig 3C) but not isoproterenol (Fig 3C) reduced the amplitude of the capsaicin currents. Moreover, we examined the effects of the specific antagonists on the inhibitory effects of clonidine. Yohimbine (1 nM) was applied in combination with clonidine (Fig 3B). The control responses of clonidine in the absence of yohimbine were recorded from the neurons in the same cultures. The inhibition of capsaicin currents by clonidine was reversed by yohimbine, suggesting that α_2_ adrenoceptors contributed to the inhibition of capsaicin currents.

### Intracellular mechanisms of the suppression of the capsaicin current by NA

**Fig 4:**
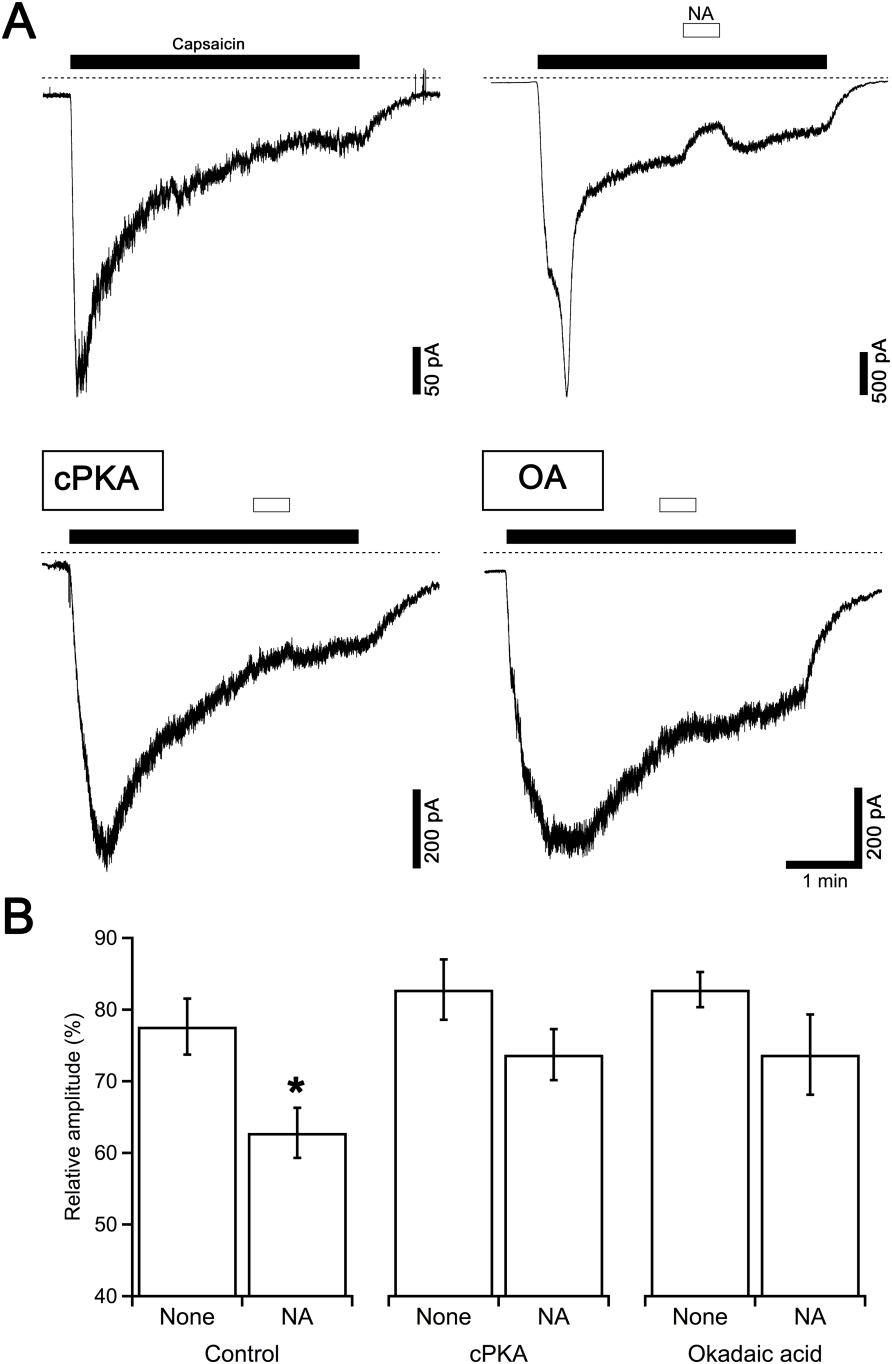
Effects of cPKA and OA on the NA-induced suppression of capsaicin currents. (A) Typical control current responses to capsaicin (1 μM) applied for 4 min are shown in the upper left panel. NA (1 nM) was applied 2 min after the beginning of the capsaicin application for 30 s. Current traces in the lower panels were recorded from the neurons dialyzed with the pipette solution containing cPKA (250 U/ml, left) and OA (1 μM, right) in the same protocol. Dashed lines indicate zero current level. (B) The relative amplitudes of the capsaicin currents at the end of the NA application compared to those just before the application of NA are shown. The columns and vertical bars indicate the mean ± SEM (n = 12, 33, 5, 11, 8 and 10 from the left). * P < 0.05 against the control data in the absence of NA (none).

All the subtypes of adrenoceptors are G protein-coupled receptors. α_2_ receptors are coupled with G_i/o_-type G protein, and the activation of the receptor causes the suppression of adenylate cyclase (AC) activity. In contrast, β receptors are coupled with G_s_-type G protein and enhance AC activity. The increased intracellular cAMP concentration activates cAMP-dependent protein kinase (protein kinase A, PKA) and the decreased cAMP concentration deactivates PKA. To determine how the modulation of AC activity regulates capsaicin-evoked currents, we examined the effects of the catalytic subunits of PKA (cPKA) and the phosphatase inhibitor, okadaic acid (OA), on capsaicin currents. cPKA and OA were applied to the pipette solution. In the presence of intracellular OA (1 μM), NA itself evoked an unidentified inward current. Because of this response to NA, an assessment of the effects of NA on the capsaicin current by the same protocol as Fig 2 was difficult. Therefore, the effects of NA on inward current responses during the prolonged application of capsaicin in the presence and absence of intracellular cPKA and OA were examined. Capsaicin applied for 4 min generated current responses with an initial rising phase (–75 ± 13 pA/pF, n = 45) followed by a sustained phase that gradually decreased by 58 ± 3% (n = 45) over 2 min. The application of NA for 30 s was started 2 min after the beginning of the application of capsaicin. In the control neurons with intracellular dialysis without cPKA or OA, NA reduced the amplitudes of the capsaicin currents as shown in the upper right panel in Fig 4A, and the amplitude recovered after the removal of NA. In the presence of cPKA or OA in the intracellular solution, capsaicin evoked an inward current (–49 ± 11 pA/pF, n = 16; –29 ± 9 pA/pF, n = 18; respectively). The decreasing ratios of inward currents during the initial 2 min of capsaicin application were 55 ± 4% (n = 16, P > 0.05, cPKA) and 44 ± 5% (n = 18, P < 0.05, OA), indicating that the intracellular dialysis of neurons with OA, but not cPKA, suppressed the desensitization of TRPV1. In these neurons, NA applied for 30 s did not show an inhibitory effect on the amplitudes of the sustained capsaicin currents (lower panels in Fig 4A). Pooled data are shown in Fig 4B. The columns show the relative amplitudes of the capsaicin currents compared with those just before the application of NA. Control responses in the absence of NA (none) were recorded in the neurons in the same culture in which the effects of cPKA and OA were examined.

### Involvement of G protein activation in the suppression of capsaicin currents by NA

**Fig 5:**
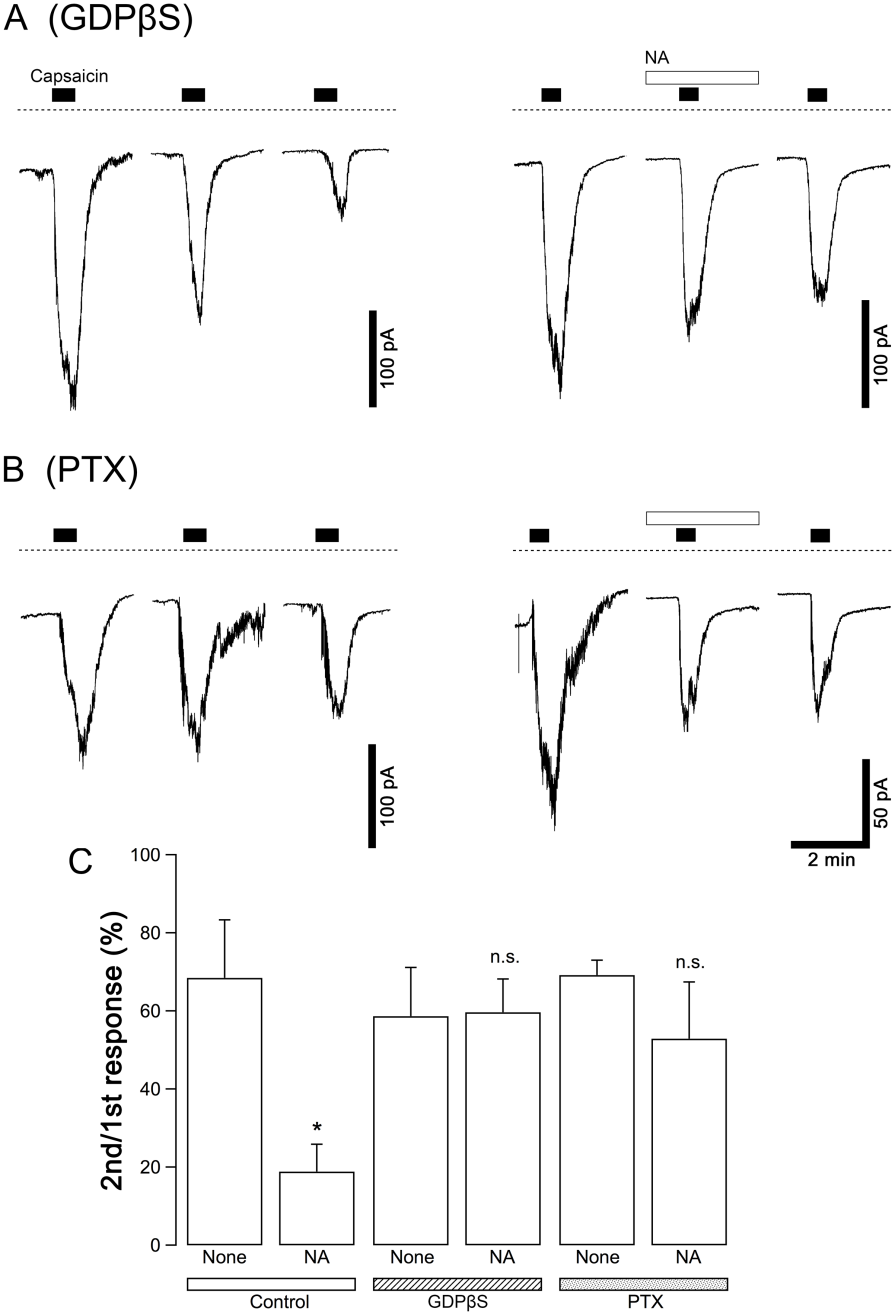
Contribution of the activation of G proteins to the NA-induced suppression of capsaicin currents. (A-B) Typical current responses in the neurons dialyzed with GDPβS (200 μM, A) and in the neurons pretreated with PTX (100 μg/ml, B). Capsaicin (1 μM) was applied for 30 s three times at intervals of 15 min to the same neuron (left panels). NA (1 pM) was applied 2 min before the second capsaicin stimulation for 4.5 min (right panels). Dashed lines in panels A and B indicate zero current level. (C) The relative peak amplitudes of the capsaicin currents by the second stimulation to those by the first stimulation in each neuron are shown (2nd/1st response). The columns and vertical bars indicate the mean ± SEM (n = 5, 8, 5, 6, 5 and 5 from the left). * P < 0.05 against the control data in the absence of NA (none).

To determine whether G protein activation contributes to the suppression of the capsaicin current by noradrenaline, the effects of intracellular dialysis with GDPβS, a non-hydrolysable GDP analog that deactivates all subtypes of G proteins, and pretreatment with pertussis toxin (PTX), which deactivates G_i/o_ proteins, were examined. GDPβS was simply supplemented in the pipette solution at a final concentration of 200 μM. PTX (100 μg/ml) was applied to the culture medium, and the neurons were cultured with PTX for 24 h before recording the current responses. Capsaicin was applied 3 times to each neuron in the same protocol as Fig 1, and the second stimulation was applied in the presence of NA. The control responses without NA were observed in the neurons in the same cultures in which GDPβS and PTX were applied. The relative amplitudes of the current responses to the second stimulation with capsaicin to those of the first were compared between the control neurons and the tested neurons. In both the neurons dialyzed with GDPβS and the PTX-treated neurons, NA did not affect the amplitude of the capsaicin current: GDPβS and PTX inhibited the inhibitory effect of NA on capsaicin currents (Fig 5).

## Discussion

### NA inhibits TRPV1 channels activated by capsaicin in sensory neurons

The results in the behavioral experiments, in which NA was injected under the skin, suggested that peripheral adrenoceptors enhanced the pain sensation when tissues were injured or inflamed [29]. In contrast, α_2_ agonists have been reported to show analgesic effects depending on various mechanisms [30–33]. However, until recently, little has been known about the functional association between adrenoceptors and TRPV1 channels, which is one of the most important molecules in sensing noxious stimuli at peripheral sensory nerves. Very recently, the activation of α_2_-adrenoceptors has been reported to suppress the sustaining activity of TRPV1 in the presence of capsaicin depending on the activity of CAMKII [24]. In this study, we demonstrated that NA suppressed both the initial rising phase and the sustained phase of current responses to capsaicin in DRG neurons in the primary culture. We have investigated the underlying mechanisms of these effects of NA.

The responses of capsaicin-sensitive DRG neurons to NA varied greatly: capsaicin responses in some neurons had very high sensitivity to NA, but others showed no sensitivity. This may depend on the expression density of the assigned adrenoceptors in each DRG neuron. Although the p*Kd* (or p*Ki*) values of NA against any adrenoceptors are lower than 8 (see IUPHAR/BPS Guide to Pharmacology, http://www.guidetopharmacology.org), NA at lower than 1 pM abolished capsaicin currents in some neurons. These results suggest that the activation of only a small proportion of adrenoceptors is required to suppress capsaicin currents and that adrenoceptors and TRPV1 closely interact in a delimited region of the cell membranes of the DRG neuron.

### α_2_-adrenoceptors suppress TRPV1 activity

Adrenoceptors are classified into α_1_, α_2_ and β subtypes depending on the types of G proteins coupled with the receptors. α_1_ receptors are coupled with G_q_, which stimulates phospholipase C when activated. α_2_ receptors are coupled with G_i/o_, which inhibits AC. In contrast, β receptors are coupled with G_s_, which stimulates AC. All of the subtypes of the adrenoceptor are reportedly expressed in DRG tissues [19–23]. We tried to assess the effects of specific agonists and antagonists of adrenoceptors. Among these adrenoceptors, α_1_ receptors are unlikely to be involved in the inhibitory effect of NA on capsaicin currents because prazosin, an α_1_ antagonist, did not affect the inhibitory effect of NA on capsaicin currents. The activation of α_1_ receptors has been reported to potentiate capsaicin responses by reducing the desensitization of TRPV1 in the observations of the intracellular concentration of Ca^2+^ [34]. However, in the observations in this study, NA mainly showed an inhibitory effect on the capsaicin response in the whole-cell voltage clamp recordings. In some neurons, NA did not reduce capsaicin currents. In such neurons, the inhibitory effect and the potentiating effect may compete.

The results that yohimbine and propranolol antagonized the effects of NA suggest that α_2_ and β receptors are likely to be involved in the inhibitory effect of NA on capsaicin currents. α_2_ and β receptors have opposite effects on AC and the activation of PKA. Therefore, we examined the effects of the catalytic subunits of PKA and OA, a phosphatase inhibitor, on capsaicin currents to determine how the AC/cAMP/PKA pathway modulates capsaicin currents. Many proteins in the neurons dialyzed with the pipette solutions containing cPKA or OA are expected to be phosphorylated. Under these conditions, capsaicin evoked significant inward currents similarly to the control neurons. During the prolonged application of capsaicin for 4 min, the inward currents rose rapidly and then decreased gradually. This decrease is caused by the desensitization of TRPV1. In the presence of OA, the rate of decay of the sustained phase of the capsaicin current was slower than in the control neurons. This result is consistent with many reports that increased phosphorylation levels suppress the desensitization of TRPV1. For example, the activation of EP_4_ receptors by prostaglandin E_2_, which activates PKA, enhances capsaicin currents depending on the phosphorylation of TRPV1 channels [35]. TRPV1 has several phosphorylation sites for PKA. Responses to capsaicin are reportedly enhanced when either serine positioned at 116 or threonine positioned at 370 are phosphorylated by PKA [36–38]. These reports and our results indicate that the phosphorylation of TRPV1 and/or related proteins by PKA did not cause the reduced TRPV1 activity. Therefore, the typical intracellular signaling pathway following the activation of β receptors and the activation of PKA does not seem to be involved in the inhibitory effect of NA on capsaicin currents in DRG neurons.

The activation of either somatostatin or μ opioid receptors, both of which are coupled with G_i/o_ proteins, has been reported to inhibit TRPV1 activity and cause analgesic effects [39, 40]. The results that yohimbine antagonized the inhibitory effect of noradrenaline and that clonidine inhibited capsaicin currents suggest the possibility that the activation of α_2_-adrenoceptors suppresses TRPV1 activity, and this effect of NA is expected to have a role in pain relief. In addition, intracellular dialysis with GDPβS and the pretreatment of neurons with PTX blocked the effects of NA, strongly suggesting that the activation of α_2_-adrenoceptors and the subsequent activation of G_i/o_ proteins are involved in the inhibitory effect of NA on capsaicin currents.

Recently, the activation of α_2_-adrenoceptors has been reported to rapidly suppress TRPV1 activity. NA and clonidine inhibit the sustained phase of capsaicin currents [24], and this inhibitory effect depends on CAMKII activity, such as the actions of D1 and D5 dopaminergic receptors on TRPV1 channels [41]. We also examined effects of KN-93, a CAMKII inhibitor, on the inhibitory effect of NA on the capsaicin current. KN-93 showed no effect in our preparations of cultured DRG neurons. It is not clear why the preparations in the study by Chakraborty *et al*. and those in this study showed different responses to KN-93. It might be caused by some differences in the procedures of isolation of neurons and/or in the condition of the cell culture. We consider that the PKA-dependent cascade may contributes to the inhibitory action of NA on TRPV1 activation, in addition to the CAMKII-mediated mechanism proposed by Chakraborty *et al*. NA may show an inhibitory effect on capsaicin currents depending on the dephosphorylation of TRPV1 channels and/or related proteins in DRG neurons through the inhibition of the activity of AC following the activation of α_2_-adrenoceptors.

### How does NA exert analgesic effects *in vivo*?

Based on the evidence that capsaicin-evoked miniature EPSPs recorded from the secondary neurons in the dorsal horn of the spinal cord were inhibited by clonidine, Chakaraborty *et al*. proposed that the inhibition of TRPV1 by α_2_ receptors resulted in the suppression of synaptic transmission from primary to secondary neurons [24]. The sustained phase of capsaicin currents is considered to include a desensitization-resistant component that is observed when TRPV1 channels are chronically activated. Therefore, noradrenergic inhibition at presynaptic terminals may play a role in the relief of chronic pain caused by the chronic activity of TRPV1 channels at the central terminal of the primary afferent. However, chronic activities of TRPV1 channels on cell bodies and nerve fibers may cause action potentials that lead to synaptic transmission to secondary neurons. Once action potentials are generated in primary afferents and conducted to central terminals, voltage-gated Ca^2+^ channels are activated and Ca^2+^ influx into the nerve terminals occurs. Since this Ca^2+^ influx causes neurotransmitter release, NA is unlikely to inhibit synaptic transmission depending on action potentials and shows analgesic effects by acting on TRPV1 channels in the central terminals. NA has been reported to have analgesic effects when applied not only systemically but also peripherally [30, 31], suggesting that another mechanism is involved in NA-induced analgesia.

We have shown that the pretreatment of neurons with NA inhibits the initial phase of the activation of TRPV1 by capsaicin and hypothesize that NA reduces the pain sensation by inhibiting TRPV1 activity before evoking action potentials at the peripheral sensory nerves. In this effect of NA on TRPV1 activation, PKA-dependent mechanisms may play a role in modulating the sensitivity to various noxious stimuli in the peripheral nerves. Since we only observed responses to capsaicin in the cell body in this study, the effects of NA on TRPV1 activated by other types of stimuli should be investigated. Moreover, a histological analysis to clarify the spatial distribution of TRPV1-positive primary afferent fibers and noradrenergic fibers under the skin is necessary in future studies to verify the physiological interaction between adrenoceptors and TRPV1 in the peripheral nerves of nociceptive neurons.
